# How Emotions Influence Cognitive Control: A Within-Subject Investigation

**DOI:** 10.3390/bs16010089

**Published:** 2026-01-08

**Authors:** Tristan Feutren, Ludovic Fabre

**Affiliations:** Centre de Recherche de l’École de l’Air (CREA–UR 09.401) École de l’air et de l’espace—Base Aérienne 701, F-13661 Salon Air, France

**Keywords:** emotions, cognitive control, inhibition, updating, shifting

## Abstract

This study examined how negative emotions influence three core components of cognitive control, inhibition, updating, and shifting, as assessed through a Go/No-Go, 2-back, and set-switching task, respectively. Participants performed these three tasks under both negative and neutral emotional conditions. Negative emotions led to slower response times on false-positive trials, suggesting increased interference during inhibitory demands rather than a direct impairment of inhibition. In the 2-back task, accuracy decreased on Non-Match trials under negative emotions, indicating difficulties in updating working memory and disengaging from irrelevant information. In the switching task, participants showed higher error rates under negative emotions regardless of trial type, pointing to a broader decline in performance when cognitive flexibility is required. Correlation analyses indicated that emotion-related effects were associated between updating and shifting, but not with inhibition, suggesting that negative emotions preferentially affect partially overlapping control processes depending on their cognitive demands. These findings highlight that the impact of negative emotions is not uniform across executive functions and underscore the importance of investigating emotion–cognition interactions across multiple domains within individuals.

## 1. Introduction

The influence of negative emotions on cognition has been documented across various domains, from fundamental processes like attention, memory, reasoning, and decision-making (for reviews, see Lemaire, 2021; M. D. Robinson et al., 2013), to more specific tasks such as arithmetic problem-solving (e.g., Fabre & Lemaire, 2019; Melani et al., 2025) and time estimation (e.g., Droit-Volet & Gil, 2009; Droit-Volet & Meck, 2007; Gil & Droit-Volet, 2011). Indeed, emotions have been shown to either impair performance (e.g., Dresler et al., 2009; Keefe et al., 2019), enhance performance (Hsieh & Lin, 2019; Phelps et al., 2006; O. J. Robinson et al., 2013), or exert no measurable effect on behavioral outcomes (for a review see Lemaire, 2021). Beyond these broad effects, a longstanding question concerns how emotions interfere with cognitive processing. Most theoretical hypotheses on the emotions effect converge on the idea that the emotions effect arises from interactions between attentional mechanisms and domain-specific cognitive mechanisms (Blanchette et al., 2014; Blanchette & Caparos, 2013; Caparos & Blanchette, 2017; Ellis & Ashbrook, 1989; Ellis & Moore, 1999; Okon-Singer et al., 2015; Padmala et al., 2011; Pessoa, 2009; Pratto & John, 1991; Yiend et al., 2013). For example, Pratto and John (1991) assume that negative content automatically draws attention through a vigilance mechanism. Pessoa (2009) also posits that, due to limited cognitive capacity, emotions tax resources, thereby reducing those available for executive demands. Although these accounts differ in emphasis, they share the assumption that the emotions effect arises from a general mechanism of attentional capture, while leaving unspecified which specific mechanism of attentional control is disrupted. Yet, recent findings indicate that emotions do not impair all components of the same cognitive function uniformly. Instead, they appear to affect specific components (e.g., Melani et al., 2025). This gap motivates this study, which aimed to investigate how emotions influence the three core components of cognitive control: inhibition, updating, and shifting. Although previous studies showed that negative emotions influence cognitive control performance, it remains unclear whether negative emotions modulate all executive processes equally, or whether they selectively disrupt specific mechanisms. To address this issue, the present study adopts a within-subject design, which allows for a direct comparison of the emotions effect across distinct control processes and provides a more integrated understanding of how negative emotions influence cognition.

Cognitive control is the ability to guide behavior according to internal goals by focusing on relevant information and ignoring distractions (e.g., Gratton et al., 2018). It helps reduce interference and supports flexible adaptation to changing demands (Banich et al., 2024). Cognitive control is generally thought to rely on three core executive processes: inhibition, updating, and shifting (Miyake et al., 2000; Friedman & Miyake, 2017). Inhibition refers to the ability to suppress automatic responses when they are inappropriate, often assessed with Go/No-Go or Stroop tasks. Updating involves monitoring and replacing outdated information in working memory, typically measured with n-back tasks. Shifting reflects the capacity to flexibly switch between task sets or response strategies and is usually evaluated through task-switching paradigms. Despite growing interest in emotion and cognitive control interactions, and although previous studies have examined emotional influences on executive control, no study has examined how negative emotions influence their three core components within the same group of participants. The present experiment adopts a within-subject design to directly compare how emotional states modulate each of these control mechanisms. Before presenting the rationale and methods of the current study, we briefly review prior findings on how negative emotions affect performance in each of these domains.

### Emotions Effect on Cognitive Control

The influence of emotions on cognitive control has been widely explored using tasks that embed emotional stimuli directly within paradigm assessing inhibition (Hare et al., 2005; Myruski et al., 2017; Schulz et al., 2007), updating (Kopf et al., 2013; Ladouceur et al., 2009; Levens & Gotlib, 2010), or shifting (Eckart et al., 2023; Kraft et al., 2020; Reeck & Egner, 2015). A major limitation of this approach is that emotions are inherently task-relevant, limiting the assessment of incidental emotional influences on cognitive control processes. Although previous studies have demonstrated that negative emotional stimuli can capture attention and modulate motor behavior when they are relevant to the task (e.g., Mirabella & Montalti, 2025; Mirabella et al., 2024), no study to date has reported comparable effects using IAPS pictures that are entirely task-irrelevant and induced prior to each trial. The present study adopts an emotional induction approach, whereby emotions were manipulated through the presentation of affective images drawn from the International Affective Picture System—IAPS (Lang et al., 1999) before each trial. This design allows emotions to be induced while keeping the cognitive task content neutral.

Although the influence of emotions on cognitive control has been investigated, findings remain inconsistent. In inhibition tasks using the Go/No-Go paradigm, results are mixed (e.g., Albert et al., 2010; Buodo et al., 2017; De Houwer & Tibboel, 2010; Kalanthroff et al., 2016; Krompinger & Simons, 2009; Myruski et al., 2017; Ocklenburg et al., 2017; Stockdale et al., 2020). Albert et al. (2010) reported increased commission errors under negative emotions, attributing this to motivational interference (e.g., approach/avoidance tendencies). Similarly, Kalanthroff et al. (2016) found faster Go responses but more No-Go errors under negative emotions, suggesting attentional capture disrupts inhibitory control. The literature also exhibits mixed findings concerning the influence of emotions on updating (e.g., Buratto et al., 2014; Grissmann et al., 2017; Ozawa et al., 2014). Buratto et al. (2014) observed enhanced delayed recognition for items encoded under negative emotions in an n-back task, indicating that emotional salience may support memory consolidation under load. In contrast, Grissmann et al. (2017) found that negative emotions impaired both accuracy and speed in a high-load n-back task, likely due to attentional resource competition. For shifting, only two studies have addressed emotional influences. Demanet et al. (2011) showed that high-arousal stimuli, regardless of valence, disrupted task-switching performance. Conversely, Wang et al. (2017) reported that positive emotions reduced switch costs, suggesting enhanced cognitive flexibility.

In sum, previous findings on emotional influences across cognitive control processes remain inconsistent, partly due to methodological differences in induction format (trial vs. block), timing (embedded vs. pre-task), and stimulus characteristics (valence, arousal). This lack of clarity in the literature regarding the respective contributions of valence and arousal motivated our decision to focus specifically on emotional valence by selecting negative emotions only. Indeed, negative emotions are known to elicit a robust attentional bias (e.g., Öhman et al., 2001; Carretié, 2014) and to be processed in a prioritized manner, making them particularly suitable for investigating how emotions modulate attentional allocation. Consequently, throughout the present study, the term “emotions effect” is used to refer to the ability of emotional stimuli to transiently capture attentional resources and compete with task-related processing. Moreover, most studies assessed a single process using between-subject designs, limiting cross-process comparisons. To overcome these limitations, the present study used a within-subject design (e.g., Hartanto et al., 2020), allowing direct comparison of emotional effects on inhibition, updating, and shifting. This approach clarifies whether negative emotions exert a general or selective impact on executive functions. This study also adopted a standardized, trial-level emotional induction with IAPS pictures to examine how transient emotional events momentarily capture attentional resources, without aiming to induce a sustained emotional state that could generate carryover or habituation effects. In addition to methodological limitations related to emotional induction procedures, certain gaps remain in the literature. First, Leblanc-Sirois et al. (2018) emphasized the relevance of false-positive response times (RTs) (i.e., RTs on No-Go trials), an often-overlooked indicator of failures in inhibitory control. Yet, to date, no study has explored how emotional states affect these false-positive RTs. Second, few studies have systematically examined whether negative emotions selectively impact distinct subcomponents of working memory, such as updating versus maintenance. To address these issues, the present study investigates how negative emotions modulate performance in a Go/No-Go task, with particular attention to false-positive RTs, and in a 2-back task designed to isolate updating processes.

Above and beyond replicating previous findings showing interference effects under neutral emotions on each task involving inhibition (e.g., Albert et al., 2010; Kalanthroff et al., 2016), updating (e.g., Buratto et al., 2014; Grissmann et al., 2017), and shifting (e.g., Demanet et al., 2011; Dreisbach & Goschke, 2004; Wang et al., 2017), the present data were collected to test the emotions effect on cognitive control processes. We hypothesized that negative emotion processing interferes with cognitive control, but not uniformly across all processes. Instead, we expected selective disruptions depending on the executive mechanism involved. For inhibition, we predicted that negative emotions would alter interference resolution, leading to larger commission errors and slower responses on No-Go trials. For updating, we expected performance to decline under negative emotions specifically on Non-Match trials, with longer latencies and lower accuracy, due to difficulty disengaging from negative emotions and replacing outdated information in working memory. For shifting, we anticipated that Switch trials would be more sensitive to negative emotions than Repeated trials, reflected in slower and less-accurate responses, indicating impaired task-set reconfiguration. Finally, we predicted that the emotions effect would correlate across tasks, especially between updating and shifting, which both rely on dynamic attentional control and flexible cognitive processing.

## 2. Materials and Methods

### 2.1. Ethics Statement

This experiment received approval from *Comité de Protection des Personnes Sud-Est-IV in France* (Ref #: 2023-A01670-45).

### 2.2. Sample Size

Following previous studies on the influence of emotions on cognitive control processes (Thompson et al., 2022), where effect size of emotion × trial type interactions was 0.48, we used a conservative estimate η^2^_p_ = 0.3, with an *alpha level* of 0.1 and a 2 × 2 repeated-measures design (emotion and trial type). The analysis indicated that a minimum of 29 participants would be required to achieve 90% statistical power. We recruited 41 participants from the French Air Force Academy (14 females, *mean age*: 24.4 years; *range*: 19–33 years) to exceed this criterion.

### 2.3. Emotional Pictures

Nine hundred and twelve pictures were selected from the International Affective Pictures System (IAPS, Lang et al., 1999). Half of the pictures depicted negative events and the other half depicted neutral events. Negative and neutral images were balanced in terms of number and systematically distributed across tasks and trial types, ensuring that each condition contained comparable proportions of emotional and neutral stimuli and preventing systematic biases across the experimental design (see Table A1 in the Appendix A). Negative images primarily depicted aversive scenes (e.g., threat- or injury-related content, such as mutilation, for example), whereas neutral images consisted mainly of everyday objects or non-emotional scenes (e.g., ironing board).

### 2.4. Design

The PsychoPy-software 3.8.10 (Peirce et al., 2019) controlled stimulus presentation, response recording, and collection of RTs with 1-millisecond accuracy. Participants were individually tested in a single session lasting approximately 60 min. Each session comprised three cognitive tasks: a Go/No-Go task, a 2-back task, and a set-switching task. The order of tasks was randomized across participants.

#### 2.4.1. Go/No-Go Task

**Figure 1:**
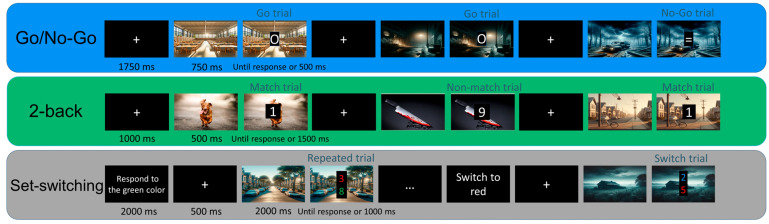
Flowchart for Go/No-Go, 2-back and set-switching task. The numbers shown in the flowchart represent the task stimuli. In the 2-back task, participants judged whether the current number matched the one presented two items earlier, whereas in the set-switching task, they determined whether the number displayed in the target color was odd or even.

The task consisted of four blocks of 96 randomly distributed trials (72 Go and 24 No-Go trials). Two distinct stimuli were used: a white “O” for Go trials, and a white “=” for the No-Go trials. Stimuli were presented at the center of a black square and measured approximately 14% of the screen height. Each trial began with a fixation cross displayed for 1000 ms, followed by an emotional picture (either neutral or negative) presented for 1000 ms. Subsequently, the stimulus was superimposed on the emotional picture and remained on screen until the participant responded or for a maximum of 500 ms. Participants were instructed to press the right arrow key of the keyboard as quickly as possible for the Go trials and to withhold their response to No-Go trials. Correct-response RTs (Go trials), false-negative rate (percentage of omissions on Go trials), false-positive RTs (No-Go trials), and false-positive rate (percentage of commissions on No-Go trials) were analyzed. A schematic illustration of the trial structure is described in Figure 1.

#### 2.4.2. Two-Back Task

This task consisted of numerical digits (1–9) displayed in white font over a black square, with a height of approximately 10% of the screen. The ratio of odd to even numbers was counterbalanced across trials. Half of the participants were instructed to press the “L” key on a keyboard whenever the current number matched the one presented two trials earlier (i.e., Match trials), and to press the “S” key when there was no match (i.e., Non-Match trials). The other half received the inverse mapping. The experiment included four blocks of 72 trials (24 Match and 48 Non-Match trials). Half of the trials were presented under neutral emotions, while the remaining trials were presented under negative emotions. Each trial began with a fixation cross displayed for 1000 ms, followed by an emotional picture presented for 1000 ms. Then, the number was superimposed on the picture for 1500 ms during which participants could respond. Response times and error rates for each trial type were recorded.

#### 2.4.3. Set-Switching Task

Participants had to respond whether a target digit number stimulus was odd or even. In each trial, two digits (one odd and one even) were displayed one above the other in distinct colors. Participants were instructed to respond based on the number presented in the target color, as indicated at the beginning of each block (e.g., “Respond to green”). Three rule-switch trials occurred per block (at Trials 8, 17, and 24), during which a new color became the target, while the previous target color became the distractor. Response mapping was counterbalanced across participants: half were instructed to press the “L” key for even numbers and the “S” key for odd numbers, and the other half the opposing mapping. Moreover, the position of both odd and even numbers was counterbalanced. They completed eight blocks of 30 trials. Each block consisted of 27 Repeated and 3 Switch trials; one half were presented under neutral emotions, and the other half under negative emotions. Each trial started with a fixation cross (1000 ms), followed by an emotional picture (1000 ms). Then, the digit stimuli were presented for 1000 ms, superimposed on the emotional picture. Only the four Repeated trials preceding and following a Switch trial were included in the analyses of RTs and error rates.

### 2.5. Data Analysis

We first examined whether negative emotions influenced performance as a function of trial type in each task (i.e., Go vs. No-Go; Match vs. Non-Match; and Repeated vs. Switch trials). Mean RTs and error rates were analyzed using 2 (trial type: within-subject) × 2 (emotion: neutral, negative) repeated-measures analyses of variance (ANOVAs). All analyses followed a hierarchical omnibus-first approach, with Holm–Bonferroni–corrected post hoc tests.

To further assess whether the impact of emotions on cognitive control was consistent across tasks, we computed emotions effect scores for each trial type and each dependent variable (RTs and accuracy). These scores were calculated as normalized change scores using the following formula:

$$Emotions\;Effect\;(\%)=\frac{PerformanceNegative-PerformanceNeutral}{PerformanceNeutral}$$

This formula yielded an individual-level percentage score reflecting the relative impact of negative emotions compared to the neutral condition. Separate scores were computed for each trial type (Go, No-Go, Match, Non-Match, Repeated, and Switch). We then conducted Pearson correlation analyses across tasks to assess whether individuals who exhibited stronger emotion-related effects in one task showed similar patterns in the other tasks. Finally, additional repeated-measures ANOVAs were conducted on the normalized emotions effect scores to examine systematic differences in the magnitude of emotions effect between tasks and trial types.

## 3. Results

### 3.1. Go/No-Go Task

**Figure 2:**
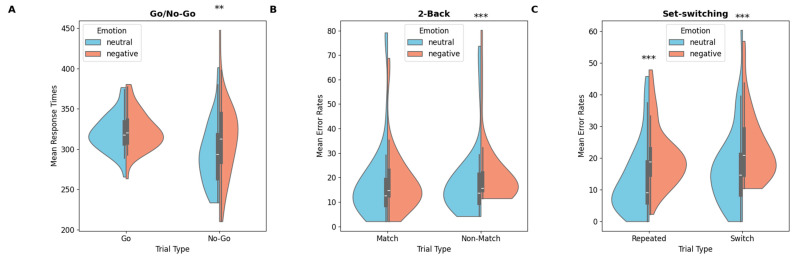
Violin plots of mean response times (Go/No-Go) and mean error rates (2-back, set-switching) for neutral and negative emotional conditions: (**A**) Go/No-Go, (**B**) 2-back, (**C**) set-switching (*p* < 0.01 **; *p* < 0.001 ***).

Response times and accuracies were analyzed using a 2 (emotion: neutral; negative) × 2 (trial type: Go; No-Go) repeated-measures ANOVAs. Results are summarized in Table 1 and the distribution of performance across emotional conditions is shown in Figure 2. Confidence intervals are presented in Table A2.

#### 3.1.1. Response Times

A significant main effect of trial type was observed, *F*(1,40) = 34.5, *p* < 0.001, MSe = 15,283, η^2^_p_ = 0.46. Participants were slower to respond on correct-Go trials than on false-positive trials (322 ms vs. 302 ms, respectively). Analyses revealed a main effect of emotion, *F*(1,40) = 10.00, *p* = 0.003, MSe = 3874, η^2^_p_ = 0.20, qualified by a significant emotion × trial type interaction, *F*(1,40) = 5.65, *p* = 0.022, MSe = 2302, η^2^_p_ = 0.12. Holm–Bonferroni-corrected post hoc comparisons showed a significant difference, *t*(40) = 2.82, *p* = 0.030, Cohen’s *d* = 0.44, with longer RTs for false-positive trials under negative emotions (311 ms) compared to neutral emotions (294 ms).

#### 3.1.2. Percentages of Errors

Analysis revealed a significant main effect of emotion, *F*(1,40) = 6.08, *p* = 0.018, MSe = 0.01, η^2^_p_ = 0.13. Participants made more errors under negative emotions compared to neutral ones (8.35% vs. 7.15%). No other main effects or interactions reached significance.

#### 3.1.3. Emotions Effect Scores

Analysis revealed a significant difference effect between correct-Go and false-positive trials on RTs, *F*(1,40) = 6.09, *p* = 0.018, MSe = 0.06, η^2^_p_ = 0.13. Participants showed a larger emotional effect on false-positive trials (+6.54%) compared to correct-Go trials (+0.92%). No significant differences in emotions effect came out significant for accuracy.

### 3.2. Two-Back Task

Participants’ mean latencies and percentages of errors were analyzed with a 2 (emotion: neutral; negative) × 2 (trial type: Match; Non-Match) repeated-measures ANOVAs (see means in Table 1).

#### 3.2.1. Response Times

A significant main effect of trial type was observed, *F*(1,40) = 50.5, *p* < 0.001, MSe = 121,431, η^2^_p_ = 0.56. Participants were slower to respond to Non-Match trials (671 ms) compared to Match trials (594 ms) under neutral emotions. This result replicates the classical effect reported in 2-back paradigms (e.g., Chen et al., 2008; Li et al., 2024).

#### 3.2.2. Percentages of Errors

A main effect of emotion resulted significant, *F*(1,40) = 9.33, *p* = 0.004, MSe = 0.02, η^2^_p_ = 0.20, qualified by a significant emotion × trial type interaction, *F*(1,40) = 5.25, *p* = 0.027, MSe = 0.01, η^2^_p_ = 0.12. Post hoc analysis revealed that participants made more errors in Non-Match trials under negative emotions (21.6%) than under neutral emotions (18.2%), *t*(40) = −5.67, *p* < 0.001, *d* = 0.88. No significant difference in accuracy was found for Match trials.

#### 3.2.3. Emotions Effect Scores

A significant difference between trial types in percentages of errors was found, *F*(1,40) = 5.40, *p* = 0.025, MSe = 0.07, η^2^_p_ = 0.12. The emotions effect was larger in Non-Match trials (−4.12%) compared to Match trials (−2.13%). No significant emotions effect was observed on RTs.

### 3.3. Set-Switching Task

Participants’ mean latencies and error rates were submitted to a 2 (emotion: neutral; negative) × 2 (trial type: Repeated; Switch) repeated-measures ANOVAs (see means in Table 1).

#### 3.3.1. Response Time

A significant main effect of trial type was observed, *F*(1,40) = 8.35, *p* = 0.006, MSe = 2102.4, η^2^_p_ = 0.17. Participants were slower to respond on Switch trials (614 ms) than on Repeated trials (607 ms). Analyses also revealed a main effect of emotion, *F*(1,40) = 6.57, *p* = 0.014, MSe = 2049.6, η^2^_p_ = 0.14. Participants were faster to respond under negative emotions (607 ms) than under neutral emotions (614 ms). No significant differences in RTs were observed between the neutral and negative emotion conditions, either for Repeated or Switch trials.

#### 3.3.2. Percentages of Errors

The analysis revealed a significant main effect of trial type under neutral emotions, *F*(1,40) = 10.6, *p* = 0.002, MSe = 0.03, η^2^_p_ = 0.21. Participants made more errors in Switch trials (17.00%) compared to Repeated trials (13.2%; for same results see Rogers & Monsell, 1995). A significant main effect of emotion was also observed, *F*(1,40) = 33.09, *p* < 0.001, MSe = 0.05, η^2^_p_ = 0.45, indicating that participants made more errors under negative emotions (22%) than under neutral emotions (15.1%). Planned comparisons revealed that participants made significantly more errors under negative emotions compared to neutral ones in both Switch trials, *F*(1,40) = 19.9, *p* < 0.001, MSe = 0.08, η^2^_p_ = 0.33, (23.5% vs. 17.0%) and Repeated trials, *F*(1,40) = 19.8, *p* < 0.001, MSe = 0.11, η^2^_p_ = 0.33 (20.5% vs. 13.2%).

#### 3.3.3. Emotions Effect Scores

No significant differences were found for either RTs or accuracy.

### 3.4. Cross-Task Correlations of Emotions Effect

**Figure 3:**
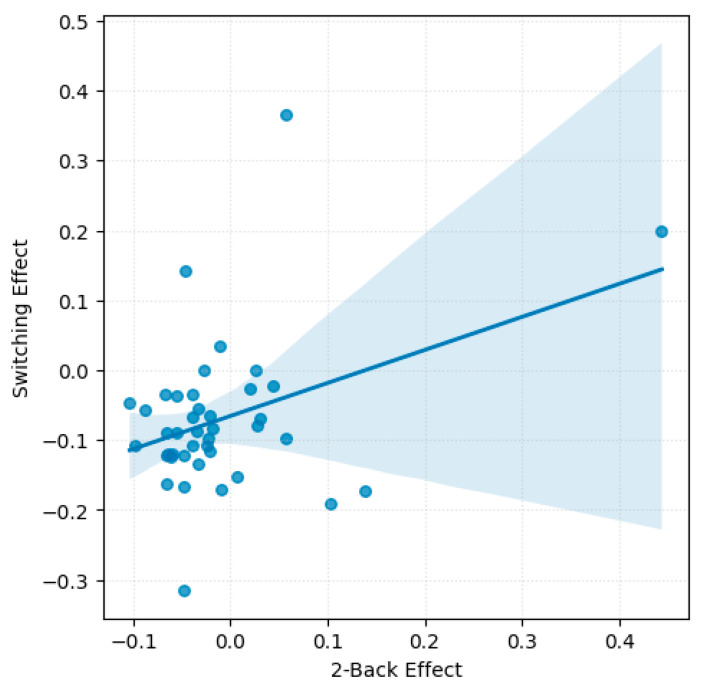
Scatterplot with regression line illustrating accuracy-based emotions effect across 2-back and set-switching tasks.

A significant moderate positive correlation emerged between the emotions effect in the 2-back task and the set-switching task, *r*(39) = 0.38, *p* = 0.014 (see Figure 3). This finding suggests that participants who showed greater emotion-related accuracy impairments in the WM task tended to exhibit similar patterns in the set-switching task. No significant correlations were found between the Go/No-Go task and either the 2-back or the set-switching task.

No significant correlations were found between emotions effect on RTs across tasks. However, a non-significant trend was observed between switching and 2-back, *r*(39) = −0.30, *p* = 0.056, consistent with the task specific analyses, which revealed no significant effect of emotions on RTs for these tasks.

## 4. Discussion

This study aimed to examine how negative emotions influence cognitive control performance. Participants completed a Go/No-Go, a 2-back, and a set-switching task. Each task was performed under both neutral and negative emotions induced via the presentation of negative and neutral pictures preceding each trial. Our results replicated well-established task-specific effects widely documented in the literature. In the Go/No-Go task, false-positive RTs were shorter than correct RTs for Go trials, consistent with previous findings (e.g., Leblanc-Sirois et al., 2018). In the 2-back task, RTs were longer for Non-Match trials compared to Match trials, as previously reported (e.g., Chen et al., 2008; Li et al., 2024). In the set-switching task, the larger percentage of errors in Switch trials compared to Repeated trials reflected the expected switching cost typically observed in cognitive flexibility paradigms (e.g., Rogers & Monsell, 1995). Interestingly, our findings revealed that negative emotions modulated performance across all three tasks, although these effects were specific to certain trial types rather than generalized across all trials. Negative emotions modulate executive processes depending on task demands. Surprisingly, in the set-switching task, negative emotions appeared to enhance overall performance, as participants responded faster under negative emotions than under neutral conditions, irrespective of trial type. Although no differences were observed in RTs between Repeated and Switch trials, such a difference emerged for accuracy, indicating that the influence of negative emotions was more pronounced on error rates than on latencies. Interestingly, the emotions effect differed across the three cognitive control tasks. Possibly, negative emotions do not influence all cognitive control processes but rather influence specific mechanisms. Furthermore, the correlation between interference effects in updating and shifting tasks could reflect an overlapping mechanism through which negative emotions influence these components of cognitive control. Taken together, these results offer important insights into the complex interplay between emotion and cognition. By highlighting both global and mechanism-specific effects of emotions on cognitive control, this study contributes to enhance our understanding on how negative emotions influence cognition.

### 4.1. Effect of Emotions on Inhibition, Updating, and Shifting

In the Go/No-Go task, participants were significantly slower on false-positive RTs under negative emotions compared to neutral emotions, indicating a slowdown in inhibitory control. This slowing may reflect increased cognitive load or attentional capture by negative stimuli, which, in turn hinders the deployment of inhibitory control processes. These findings are consistent with previous research showing that negative emotions interfere with attentional control and executive monitoring (e.g., Pessoa, 2009; Verbruggen & De Houwer, 2007). The pattern of shorter false-positive RTs has been interpreted as reflecting a failure to suppress prepotent responses that have already been initiated (e.g., Leblanc-Sirois et al., 2018). emotions modulated overall Go/No-Go performance, with a stronger impact observed on false-positive-Go trials, suggesting a differential emotional influence rather than a generalized inhibition deficit. This pattern likely reflects increased interference under high-control demands, where negative emotions compromise the ability to withhold prepotent responses. According to previous findings, emotions do not consistently influence Go trial performance when emotional content is irrelevant to the task (e.g., Mancini et al., 2020; Yu et al., 2014). No-Go trials require top-down control to inhibit dominant responses, making them more sensitive to interference. This interpretation aligns with recent theories suggesting that emotionally irrelevant information selectively disrupts executive processes when cognitive control demands are high (e.g., Grimshaw et al., 2018; Murphy et al., 2020; Zhang et al., 2023). It is therefore possible that the slowing of false-positive RTs under negative emotions reflects increased interference with top-down inhibition, particularly under cognitively demanding conditions. Moreover, participants made more commission errors under negative emotion conditions. Although the interaction between emotion and trial type was not significant for accuracy, the main effect of emotion nonetheless indicates that inhibition performance is modulated by negative emotions. Emotions effect scores on RTs revealed a larger emotional impact on No-Go trials compared to Go trials, reinforcing the idea that inhibitory control is particularly sensitive to negative emotions. Although our findings suggest an effect of negative emotions on Go/No-Go performance, caution is warranted in generalizing this effect to the broader construct of inhibition. The Go/No-Go paradigm primarily targets response inhibition (i.e., the ability to withhold a prepotent motor response), whereas other forms of inhibitory control such as interference control or proactive control (Friedman et al., 2008; Nigg, 2000) are not encompassed. As emphasized in recent theoretical work, inhibitory control is a multidimensional construct involving separable mechanisms across motor, cognitive, and attentional domains (Mirabella, 2023; van den Wildenberg et al., 2022). Consequently, our results should be interpretated as reflecting emotion-induced changes in motor response inhibition, rather than inhibition more broadly. Future studies could integrate complementary paradigms (e.g., Stroop, Stop-Signal, Antisaccade tasks) to further dissociate how emotions impact distinct inhibitory subcomponents.

In the 2-back task, participants were slower to respond to Non-Match trials compared to Match trials. This finding replicates the classic updating cost reported in the literature and reflects the increased cognitive demand associated with monitoring and comparing incoming stimuli in WM (e.g., Li et al., 2024). The significant emotion × trial type interaction in the percentages of errors showed that participants made more errors on Non-Match trials in negative emotional conditions, whereas no difference was found for Match trials. Emotions effect scores also confirmed that performance was influenced on Non-Match trials, suggesting that negative emotions impair updating performance specifically when task demands are high. Negative emotions may primarily affect the updating processes involved in integrating new content into working memory, monitoring stimuli presented two items earlier, and suppressing outdated information. This interpretation suggests that negative emotions influence these specific control operations rather than impairing recall accuracy per se. Non-Match trials are inherently more demanding, as they require participants to maintain the stimulus from two trials earlier, actively suppress information that is no longer relevant, and incorporate new input into WM. This interpretation is consistent with prior studies showing that updating is impaired when emotional states compete with task-relevant processing (e.g., Grissmann et al., 2017). The absence of the emotions effect in RTs, despite significant accuracy impairments, suggests that negative emotions do not necessarily slow down updating, but rather impair its efficiency and reliability. It is possible that negative emotions interfere with cognitive representations or increase the probability of response conflict, degrading the fidelity of the memory trace rather than slowing its retrieval (Mitchell & Phillips, 2007). This emotions effect may result from a competition for limited-capacity cognitive resources, with emotion processes drawing attention away from ongoing cognitive control processes. Previous hypotheses proposed that emotion processes are prioritized during cognitive processing at the expense of goal-relevant content (Desimone & Duncan, 1995; Pessoa, 2009). Under high cognitive load, such as in Non-Match trials, this prioritization could reduce the availability of attentional and memory resources required for effective WM updating and the suppression of irrelevant information.

In the set-switching task, participants made more errors in Switch trials compared to Repeated trials under neutral emotional conditions. These results replicate classical findings from the task-switching literature and reflect the greater cognitive demands associated with reconfiguring mental sets (e.g., Rogers & Monsell, 1995). Interestingly, a main effect of emotion revealed that participants responded faster under negative emotional conditions compared to neutral ones. Although this performance improvement was not predicted, it aligns with previous findings suggesting that negative emotions can heighten arousal or alertness, thereby accelerating responses (e.g., Kuhbandner & Zehetleitner, 2011; Pessoa, 2009). While these faster RTs under negative emotions may suggest improved performance, accuracy rates did not follow the same pattern. Such a dissociation raises the possibility of a speed–accuracy trade-off (Heitz, 2014), whereby participants under negative emotions may adopt a more impulsive response strategy. This shift could reflect a motivational tendency to complete tasks more quickly in order to reduce the negative emotions effect (Pessoa, 2009). In tasks with high executive demands, such as set-switching, this tendency may compromise the ability to maintain task goals or to reconfigure mental sets efficiently. One possible explanation is that participants engaged in a less-controlled processing mode, marked by reduced top-down regulation and increased cognitive impulsivity. Although participants generally responded faster under negative emotions, further analyses revealed that the emotions effect did not emerge in latency, whereas such effects were observed for accuracy. This pattern suggests that negative emotions were associated with a broader increase in error rates in this task, possibly reflecting reduced control efficiency under high task demands. Similar findings have been reported in the task-switching literature (e.g., Hsieh & Lin, 2019).

Interestingly, the increase in error rates under negative emotions is consistent with models proposing that negative emotions impair goal maintenance. For instance, Kuhbandner and Zehetleitner (2011) suggested that negative emotions increase distractibility, undermining the ability to shield task goals from interference. In the current study, such interference may have globally weakened participants’ ability to sustain task-relevant rules, whether shifting was required or not. Thus, negative emotions appear to compromise the goal representations, resulting in increased errors across both Repeated and Switch trials, rather than a specific disruption of executive reconfiguration mechanisms. An alternative but not mutually exclusive explanation is that negative emotions promote a more flexible processing mode. According to Dreisbach and Goschke (2004), two distinct modes of cognitive control can be distinguished: a shielded mode, which favors goal maintenance and resistance to distraction but limits flexibility, and a flexible mode, which facilitates cognitive reconfiguration at the cost of increased distractibility. Here, the increased errors and faster responses observed under negative emotions might reflect a shift toward this more flexible mode, allowing for quicker transitions but with reduced control over interference.

### 4.2. The Influence of Emotions Across the Three Tasks

One of the main contributions of the present study lies in the within-subject comparison of emotional effects across distinct cognitive control processes. Our results provide original evidence for a potential link between updating and shifting mechanisms under emotional influence. Specifically, a significant moderate positive correlation was observed between the emotions effect on accuracy in the 2-back and set-switching tasks. Participants who exhibited greater emotion-related impairments in WM updating also tended to show similar impairments in cognitive flexibility. This finding is particularly noteworthy given the distinct pattern of emotions effect observed for each task. In the 2-back task, negative emotions selectively impaired participants’ ability to discard no-longer-relevant information, evidenced by decreased accuracy in Non-Match trials. In contrast, performance in the set-switching task was globally impaired by negative emotions, with a general increase in percentages of errors across both Repeated and Switch trials. This dissociation indicates that negative emotions do not affect updating and shifting in a uniform manner, but rather perturb task-specific control mechanisms differently depending on cognitive demands. These findings suggest updating and shifting may rely on a partially overlapping control operations, such as goal maintenance, interference resolution, or the suppression of irrelevant mental representations, that are differentially recruited across tasks. Prior models of executive function (e.g., Friedman & Miyake, 2017) have emphasized the partially independent yet interconnected nature of cognitive control components, supported by domain-general mechanisms. Similarly, hypotheses of cognitive control suggest that processes like interference resolution, attentional allocation, and top-down monitoring play central roles across multiple executive functions (Gratton et al., 2018). The present findings are consistent with the hypothesis that negative emotions disrupt these partially overlapping processes, which may rely on shared cognitive resources, leading to impairments in tasks that require flexible updating and task-set reconfiguration.

An additional interpretation is that an overlapping mechanism may underlie the influence of negative emotions on both task-switching and WM-updating, specifically, the ability to actively maintain task goals and suppress irrelevant information. Negative emotions are known to receive prioritized processing (Pessoa, 2009), which can lead to stronger interference when cognitive control demands are high. This difficulty in filtering out emotional distractors could explain the observed performance impairments across both trial types in the switching task, as well as the greater error rates on Non-Match trials in the 2-back task, which require the active replacement of outdated information. Furthermore, the observed correlation between the emotions effect in the shifting and updating tasks suggests that participants who are more susceptible to emotional interference in one domain may experience similar deficits in the other. These findings are consistent with the idea that negative emotions may reduce control efficiency in a subset of executive processes, particularly under conditions of high cognitive demand, characterized by impaired goal maintenance and inhibitory filtering of affective interference.

However, no significant correlations were observed between the Go/No-Go task and either the updating or switching tasks. This pattern of results suggests that inhibitory control, as assessed in the present paradigm, is influenced by emotions through more domain-specific or process-specific pathways. One possible explanation is that the type of inhibition required in the Go/No-Go task relies more heavily on automatic, stimulus-driven processes, which are triggered by specific response demands and may be less sensitive to fluctuations in the emotional context across individuals. In contrast, updating and shifting require more sustained and flexible engagement of cognitive resources, making them more vulnerable to emotional interference and individual differences in emotional reactivity. As such, the absence of cross-task correlations involving the inhibition task may reflect distinct mechanisms by which emotions modulate executive functioning, with inhibition being relatively isolated from the broader, integrative control processes engaged in updating and shifting.

## 5. Conclusions

To conclude, these cross-task analyses reinforce the idea that the emotions effect on cognitive control is not uniform but instead varies depending on the nature of the executive process involved. While inhibitory control appears to operate independently, updating and shifting may share an overlapping mechanism sensitive to emotional influence, especially when cognitive demands involve actively suppressing or replacing irrelevant information. This pattern supports a componential model of executive function, in which distinct yet partially overlapping control mechanisms are differentially influenced by negative emotions.

One limitation of our experiment is that each executive process was assessed using a distinct task. Although this strategy allows for isolating the emotions effect on specific mechanisms, it may also introduce variability due to differences in task structure, difficulty, or emotional sensitivity. This variability may limit the comparability of negative emotions across cognitive control domains. Future research would benefit from developing integrated paradigms in which the three cognitive control mechanisms are assessed within a single, unified task. Such a design would offer a more ecologically valid and dynamic framework to investigate how negative emotions affect executive functions and would allow for fine-grained modeling of emotion–cognition interactions across mechanisms within the same context. It would also help disentangle whether emotional modulation targets distinct control processes independently or results from a more general disruption of top-down regulation under affective load. Another limitation lies in the use of a single task for each cognitive control process. As said below, the Go/No-Go task only allows the investigation of one specific facet of inhibition and may not reflect the influence of emotions on other inhibitory control subcomponents. Thus, while our results indicate emotional modulation of response inhibition, they should not be overgeneralized to the broader domain of inhibitory control. This observation also applies to other cognitive control processes. Indeed, although we deliberately restricted our design to a single cognitive task to assess each process and thereby reduce methodological variability, future research should aim to examine the influence of emotions on other subcomponents of cognitive control. A further limitation of the present study is that task stimuli were superimposed on emotional images, which may have introduced low-level perceptual differences between emotional conditions; although images were matched on normative properties and stimulus parameters were held constant, future studies should include non-overlapping or masked control conditions to fully dissociate emotional from perceptual influences.

Taken together, the present findings provide new insights into the differential impact of negative emotions on cognitive control. While negative emotions modulated performance across inhibition, updating, and shifting tasks, the nature and specificity of these effects varied by process. Notably, a significant association between emotion-related impairments in updating and shifting suggests the presence of an overlapping, emotion-sensitive control mechanism, possibly linked to the suppression of irrelevant information. In contrast, inhibition appeared to be influenced in a more domain-specific manner. These results reinforce the idea that negative emotions do not uniformly influence executive functioning but instead target specific cognitive mechanisms.

## Figures and Tables

**Table 1:** Mean response times (and percentages of errors) for the Go/No-Go, 2-back, and set-switching tasks in negative and neutral emotion conditions.

Task	Trial	Negative	Neutral	Negative–Neutral
Go/No-Go	Correct-Go trial (*N* = 144)	323 (7.47)	321 (6.42)	2 (1.05) *
	False-positive trial (*N* = 48)	311 (9.25)	294 (7.88)	17 * (1.37)
	False-positive–Go	12 * (−1.8)	27 *** (−1.46)	
2-back	Non-Match trial (*N* = 96)	679 (21.6)	671 (18.2)	8 (3.4) ***
	Match trial (*N* = 48)	598 (17.5)	594 (18.2)	4 (−7)
	Match–Non-Match	81 *** (4.1) *	77 *** (0)	
Set-switching	Switch trial (*N* = 18)	610 (23.5)	619 (17.0)	−9 (6.5) ***
	Repeated trial (*N* = 150)	604 (20.5)	610 (13.2)	−6 (7.3) ***
	Switch–Repeated	6 (1.2) *	9 (3.8) *	

Note. *: *p* < 0.05, ***: *p* < 0.001.

## Data Availability

The data that support the findings of this study are openly available in the Open Science Framework (OSF) at https://osf.io/zwbrg/?view_only=1c3d45f8d2484e8cae9b02f8a12240bb (accessed on 31 October 2025), reference number (ZWBRG).

## Abbreviations

The following abbreviations are used in this manuscript:

ANOVAs	Analysis of Variance
IAPS	International Affective Picture System
MSe	Mean Square Error
ms	Millisecond(s)
RT	Response Time(s)
WM	Working Memory
η^2^_p_	Partial Eta Squared

## Appendix A

**Table A1:** Emotional valence and arousal ratings for each type of trial in the three tasks.

Elements	Negative	Neutral	*F*(df1,df2)
	*Emotional Valence*		
Go trial (*N* = 144)	3.28 (1.81–4.75; 0.07)	6.30 (5.35–7.18; 0.04)	10,429 ***
No-Go trial (*N* = 48)	3.21 (1.31–4.88; 0.19)	6.29 (5.22–7.32; 0.12)	1644 ***
*F*(1,47)	0.931 (*p* = 0.194)	0.0121 (*p* = 0.913)	
Non-Match trial (*N* = 96)	3.23 (1.56–4.84; 0.10)	6.27 (5.24–7.27; 0.06)	3160 ***
Match trial (*N* = 48)	3.29 (1.59–4.82; 0.16)	6.31 (5.26–7.26; 0.10)	2477 ***
*F*(1,95)	1.81 (*p* = 0.185)	1.81 (*p* = 0.850)	
Repeated trial (*N* = 150)	3.25 (1.76–4.78; 0.07)	6.29 (5.32–7.22; 0.04)	10,178 ***
Switch trial (*N* = 18)	3.22 (1.67–7.81; 0.31)	6.27 (5.29–7.24; 0.19)	701 ***
*F*(1,17)	0.0227 (*p* = 0.882)	<0.001 (*p* = 0.985)	
	*Arousal Rating*		
Go trial (*N* = 144)	5.22 (2.03–6.96; 0.08)	4.81 (2.6–6.82; 0.08)	10.9 **
No-Go trial (N = 48)	5.31 (1.72–7.26; 0.19)	4.34 (2.51–6.34; 0.13)	15.7 ***
*F*(1,47)	0.849 (*p* = 0.400)	3.09 (*p* = 0.085)	
Non-Match trial (*N* = 96)	5.19 (2.39–7.39; 0.12)	4.55 (2.74–6.66; 0.10)	13.6 ***
Match trial (*N* = 48)	3.23 (2.69–7.34; 0.19)	4.39 (2.32–7.07; 0.16)	9.03 *
*F*(1,47)	1.83 (*p* = 0.183)	0.033 (*p* = 0.885)	
Repeated trial (*N* = 150)	5.16 (1.74–4.78; 0.08)	4.64 (5.32–7.22; 0.08)	15.4 ***
Switch trial (*N* = 18)	5.20 (1.67–4.81; 0.32)	4.26 (5.29–7.24; 0.23)	4.42 *
*F*(1,17)	0.485 (*p* = 0.496)	0.0216 (*p* = 0.885)	

Note. *: *p* < 0.05, **: *p* < 0.01, ***: *p* < 0.001.

**Table A2:** 95% confidence interval for each trial type of each task, on response times and accuracies.

Task	Trial	Negative	Neutral
Go/No-Go		*Response Times*	
	Correct-Go trial (*N* = 144)	[315, 330]	[313, 328]
	False-positive trial (*N* = 48)	[296, 326]	[281, 306]
		*Accuracies*	
	Correct-Go trial (*N* = 144)	[4.8, 10.1]	[3.8, 8.9]
	False-positive trial (*N* = 48)	[7.1, 11.4]	[5.9, 9.7]
2-back		*Response Times*	
	Non-Match trial (*N* = 96)	[679, 640]	[632, 710]
	Match trial (*N* = 48)	[565, 632]	[558, 630]
		*Accuracies*	
	Non-Match trial (*N* = 96)	[17.2, 26.1]	[13.4, 23]
	Match trial (*N* = 48)	[14, 22.4]	[12.4, 22.7]
Set-switching		*Response Times*	
	Switch trial (*N* = 18)	[594, 626]	[601, 6437]
	Repeated trial (*N* = 150)	[587, 622]	[595, 626]
		*Accuracies*	
	Switch trial (*N* = 18)	[20.1, 26.9]	[13.1, 20.9]
	Repeated trial (*N* = 150)	[17.4, 23.5]	[9.6, 16.7]
